# The Pull-Through Technique for Recanalization of Transjugular Intrahepatic Portosystemic Shunt Dysfunction

**DOI:** 10.1155/2020/9150173

**Published:** 2020-04-27

**Authors:** Si-liang Chen, Cheng-jiang Xiao, Shuai Wang, Si-yi Jin, Jian-bo Zhao

**Affiliations:** ^1^Department of Interventional Radiology, Guangdong Second Provincial General Hospital, Guangzhou, 510317 Guangdong, China; ^2^Department of Intensive Care Unit, Guangzhou Hospital of Integrated Traditional and West Medicine, Guangzhou, 510800 Guangdong, China; ^3^Internal Medicine Training Base, Guangdong Second Provincial General Hospital, Guangzhou, 510317 Guangdong, China; ^4^Division of Vascular and Interventional Radiology, Department of General Surgery, Nanfang Hospital, Southern Medical University, Guangzhou, 510515 Guangdong, China

## Abstract

**Purpose:**

To evaluate the technical efficacy and safety of the pull-through technique in recanalization of transjugular intrahepatic portosystemic shunt (TIPS) when standard transjugular approach is inaccessible.

**Materials and Methods:**

A retrospective review of patients underwent TIPS revision via the pull-through technique was performed. Transhepatic directly punctured stent was conducted if the portal vein could not be accessed via standard transjugular approach. Technical success was defined by recanalization of shunt. Clinical success was defined as bleeding interruption and ascites regression without pharmacological support. All patients were followed up by clinical evaluation and Doppler ultrasound.

**Results:**

Between January 2010 and December 2016, a total of 63 patients underwent TIPS revision, and 14 of them could not be accessed via standard transjugular approaches owing to stenosis or occlusion of the hepatic vein. The pull-through technique was successful in 13 patients, and one patient underwent parallel TIPS. No procedure-related complication was observed. One patient died of liver failure one week after the procedure. During the follow-up, three patients developed hepatic encephalopathy, and one patient developed TIPS dysfunction again and experienced variceal bleeding. The primary patency rate after TIPS revision was 92% (11/12) at 12 months.

**Conclusion:**

The pull-through technique was effective and safe for recanalization of TIPS inaccessible via standard transjugular approach.

## 1. Introduction

Transjugular intrahepatic portosystemic shunt (TIPS) has been increasingly used for the treatment of portal hypertension-related complications, especially variceal bleeding and refractory ascites. The 1-year primary patency rate is usually less than 50% when bare stents are used [1]. With the use of expanded polytetrafluoroethylene- (ePTFE-) covered stents, the long-term patency rate has significantly increased [2]. Shunt dysfunction still remains the main limitation. Standard transjugular approach is an important TIPS revision treatment for shunt dysfunction. However, sometimes it is difficult to get through the stenosis or occlusion via standard transjugular approach. Haskal and Cope firstly reported the method that combined transhepatic and transvenous approach to treat for TIPS dysfunction [3]. However, clinical experience regarding this technique is limited owing to only few relevant case reports [4–6]. The purpose of this study was to determine the efficacy and safety of the transhepatic guide wire pull-through technique in TIPS recanalization.

## 2. Materials and Methods

### 2.1. Data of Patients

This retrospective study was approved by the institutional review board. Between January 2010 and December 2016, a total of 251 patients underwent TIPS with ePTFE-covered stents (Fluency Plus, Bard, Tempe, Arizona, USA) in our department. Of these, 63 patients developed shunt dysfunction and need to undergo TIPS revision. We analyzed 14 (10 male and 4 female; mean age, 54.0 ± 12.9 years; age range, 26-71 years) of these 63 patients who underwent TIPS revision by pull-through approach because the portal vein could not be accessed via standard transjugular approaches (Table 1). TIPS indications included variceal bleeding (*n* = 12) and refractory ascites (*n* = 2). Three of 14 patients created TIPS using a single-covered stent, and 11 patients required an additional bare stent (E-Luminexx, Bard, Tempe, Arizona, USA) at the portal vein end. The primary patency time was 10.4 ± 2.9 months (range, 7-18 months). Two patients underwent prior TIPS revision via the transjugular approach, one received angioplasty and one received a bare stent implantation. All the procedures of the pull-through technique were performed after standard transjugular approaches had failed.

### 2.2. Definition of Shunt Dysfunction

Shunt dysfunction was suggested when any one of the following events was observed: (1) variceal bleeding, (2) occurrence of severe ascites, or (3) a maximum flow velocity < 50 cm/s or >200 cm/s within the shunt as demonstrated by Doppler ultrasound [7]. Suspected shunt dysfunction was confirmed by portography and a pressure measurement that showed a portosystemic pressure gradient (PPG) > 15 mmHg. The duration of time from the TIPS procedure to the first shunt dysfunction was defined as the primary patency.

### 2.3. TIPS Revision Procedures

**Figure 1:**
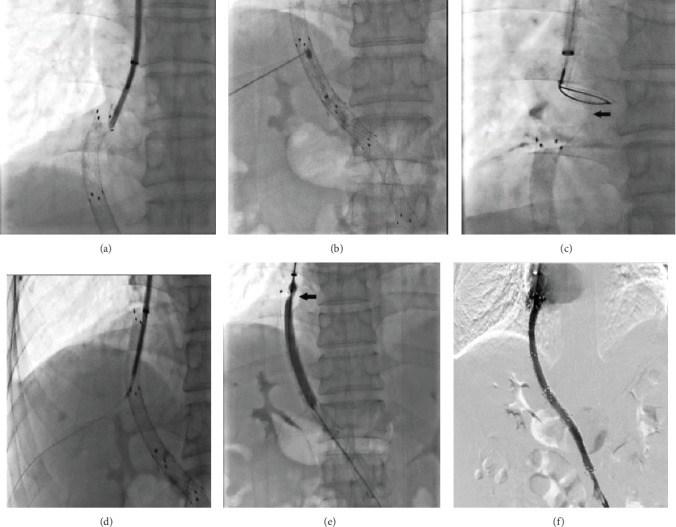
Patient No. 5 developed shunt dysfunction 18 months after TIPS. Guide wire could not access the shunt via a standard transjugular approach (a); direct puncture stent was performed, and the injected contrast medium confirmed that it reached the shunt (b); a 0.018 guide wire was advanced into RA (black arrow), and a goose-neck snare was used to capture the guide wire and pull it out from the right jugular vein (c); Flex Check-Flo introducer was advanced over the wire to get into the shunt (d); image of balloon dilated shows occlusion at the distal tip (black arrow) (e); an 8 cm covered stent was deployed, and venography shows the patency of the shunt (f).

**Figure 2:**
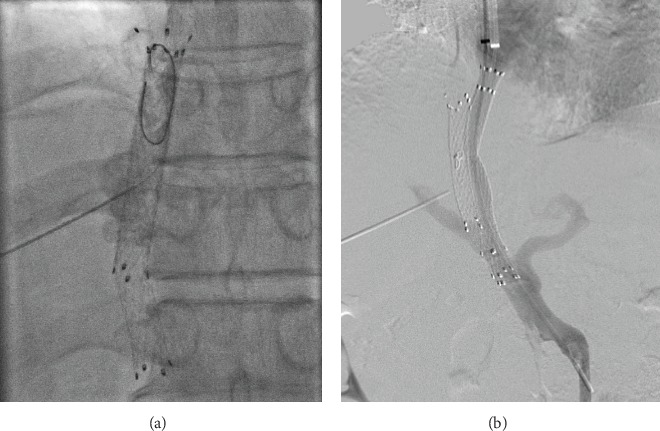
Images of pull-through technical failure. Patient No. 4 developed shunt dysfunction 11 months after TIPS. The guide wire was unable to get through the occlusion at the hepatic vein end (a). We created a parallel TIPS as an alternative (b).

The procedures were similar to previous study (Figure 1) [3]. Before attempting transhepaticly direct puncture stent, we tried to get access through the shunt via transjugular approaches. Firstly, the right internal jugular vein was accessed, following which a 10 Fr sheath of RUPS-100 set (Cook Incorporated, Bloomington, IN, USA) was advanced into the inferior vena cava (IVC) to the distal portion of the stent. Metal cannula of RUPS-100 set was advanced to the distal tip of the stent to provide support to advance the hydrophilic guide wire and 5 Fr single-curved catheter to get through the stenosis or occlusion. After it was proven inaccessible, transhepatic direct puncture was performed. Under fluoroscopic guidance, a 20-gauge needle was used to puncture into the stent via the right intercostal space in the midaxillary region; contrast medium was injected to confirm that the shunt was indeed accessed. Thereafter, a 0.018-inch guide wire was inserted via the needle and advanced into the right atrium (RA) to get through the occlusion. A goose-neck snare was introduced from the jugular sheath to capture the transhepatically placed guide wire in the RA and pulled out from the sheath to the jugular site. A 5 Fr KMP catheter (5 F, 40 cm, Cook, Bloomington, IN, United States) was advanced over the wire to get through the occlusion or stenosis at the hepatic vein end till the stent punctured the site. After withdrawing the 0.018-inch guide wire, a 0.035-inch guide wire was advanced into the superior mesenteric or splenic vein. After portal vein catheterization, portography was performed and the PPG was measured. The shunt was dilated by an 8 mm balloon (PowerFlex P3; Cordis Europa N.V., LJ Roden, The Netherlands). Next, an 8 mm stent graft in an appropriate length was deployed to cover the shunt till the junction of the hepatic vein and the IVC or occlusion area. Final shunt venography was performed, and the PPG was measured again. However, in one patient, the transhepatic 0.018-inch guide wire could not get through the occlusion at the hepatic vein end, and we hence created parallel TIPS as an alternative treatment (Figure 2).

### 2.4. Postoperative Management and Follow-Up

After successful TIPS revision, the treatment for improving liver function was regularly performed. Lactulose (10 mL, three times per day) was regularly given orally to all patients for 7 days in order to prevent hepatic encephalopathy (HE), and no further use was approved unless patients were diagnosed with HE. Anticoagulation was not routinely recommended except in patients treated for thrombosis of the hepatic veins. Antiplatelet therapy (Plavix, 75 mg, once a day, a total of 6 months) was carried out if the platelet count is more than 80 × 109/L. All patients were followed up in the outpatient clinic with clinical, biochemical, and color Doppler ultrasound evaluation, initially at 1 month after TIPS, then at 3 months, and every 6 months thereafter. The endpoint of follow-up was liver transplantation, death, end of the observation period, and/or loss to follow-up.

## 3. Results

Technical success was achieved in 13 of 14 patients (Table 2). In one patient, the transhepatically placed guide wire could not get through the occlusion at the distal tip. Therefore, we created a new parallel TIPS. PPG decreased from 22.9 ± 5.9 mmHg to 12.2 ± 2.8 mmHg (*t* = 9.97, *p* < 0.01). In seven cases, PPG less than 12 mmHg was not achieved; however, all these patients showed significant clinical benefit. A total of 14 covered stents (Fluency Plus, Bard, Tempe, Arizona, USA) and one bare stent (E-Luminexx, Bard, Tempe, Arizona, USA) were implanted. No procedure-related complications such as abdominal hemorrhage, hemobilia, or pulmonary embolism were observed. One patient died of liver failure one week after the procedure. No patient developed HE during hospitalization. During the median follow-up duration of 13.4 months (range, 0-20 months), three patients developed HE, and all were effectively controlled by medical therapy without TIPS reduction. One patient developed shunt dysfunction and experienced recurrent variceal bleeding again 3 months after TIPS revision; further revision via transjugular approach was performed, wherein stenosis at the portal vein end was confirmed, and an 8 mm bare stent (E-Luminexx, Bard, Tempe, Arizona, USA) was implanted. The remaining patients showed shunt patency with no recurrent symptoms. Therefore, the primary patency rate after TIPS revision via the pull-through technique was 92% (11/12) at 12 months.

## 4. Discussion

Shunt dysfunction is a major limitation of TIPS, and the causative factors are varied, including acute thrombosis, pseudointimal hyperplasia within the TIPS tract in the liver parenchyma, and intimal hyperplasia of the hepatic vein outflow tract. Both thrombosis and pseudointimal hyperplasia are associated with bile duct transection and biliary fistula [8, 9]. Compared with bare stent, ePTFE-covered stents can prevent bile leakage and provide a matrix for neointimal coverage of the stent surface, thereby significantly improving the patency of TIPS [10, 11].

As ePTFE-covered stents were used in TIPS, intrastent stenosis or occlusion was dramatically decreased; however, stenosis in the hepatic vein outflow majorly attributed to TIPS dysfunction. Previous studies showed that 43-100% of the stenoses found were located in the hepatic vein end of TIPS created by the Viatorr stent [10, 12]. Compared with intrastent stenosis or occlusion, it is more difficult to get through the stenosis or occlusion via standard transjugular approaches because of the stent location and angulation in relation to the hepatic vein. In the present study, 14 of 63 cases were inaccessible via purely transjugular approaches with stenosis or occlusion at the hepatic vein end. This is consistent with the results of other studies, wherein most of the cases that were inaccessible via transjugular approaches were occluded at the hepatic vein end. The transhepatic guide wire pull-through technique combined with transjugular approaches was efficient in TIPS recanalization which was inaccessible via purely transjugular approaches. In our study, 13 of 14 cases were successfully recanalized using this technique. The guide wire getting through the stenosed or occluded area is the key step in TIPS recanalization. The guide wire was much easier to pass through the occlusion at the hepatic vein end using an intrastent approach rather than from the hepatic vein or IVC. Chan and Liang reported another method to introduce the guide wire from intrastent to the IVC [13]. They recanalized an occluded TIPS via a transsplenic approach. However, this approach is more difficult for catheterization than the direct puncture technique. Moreover, it may be difficult to pass the guide wire through the occluded area owing to the long distance with less rigid scaffold. Stent punctured site near the distal tip will provide a more rigid scaffold. However, although we tried our best to puncture the stent near the distal tip to provide maximum rigidity, the guide wire still could not get through the occlusion at the hepatic vein end in one case. Creating a parallel TIPS may be a reasonable alternative in such a situation.

Consistent with previous studies, no procedure-related complications were observed [3–6]. To alleviate the injury of stent puncturing, we used a 20-gauge needle which is normally used in percutaneous transhepatic cholangial drainage, and we introduced a 0.018-inch guide wire via the needle to avoid introducing sheath for exchanging the 0.035-inch guide wire. It has been demonstrated that stent placement achieves better patency rates after TIPS revision than pure angioplasty [14]. To prevent bile leakage from the stent puncture site, we deployed an ePTFE-covered stent. In the present study, the primary patency after TIPS revision was 92% at 12 months, which is consistent with other studies [15, 16].

Some limitations of our study are its retrospective design, small sample size, and limited follow-up duration. However, this study adds to the experience of transhepatic guide wire pull-through technique in TIPS recanalization that has been limited to only a few cases reports.

In conclusion, the transhepatic guide wire pull-through technique is an effective and safe method and a reasonable alternative in TIPS inaccessible via the standard transjugular approach.

## Figures and Tables

**Table 1:** Clinical characteristics of the patients.

No.	Age/sex	TIPS indication	Etiology	Primary patency (months)	Symptoms of TIPS dysfunction	Stent type	Multiple revision	Previous ascites	Previous HE	Serum albumin (g/L)	Serum bilirubin (mmol/L)	Serum creatinine (mmol/L)	PT (s)	Child-Pugh score
1	26/F	Ascites	BCS	7	Ascites	Covered+bare	No	Yes	No	33.2	33.5	67	17.2	8
2	67/M	Variceal bleeding	HBV	11	Bleeding	Covered+bare	Yes^∗^	No	No	30	21.6	45	15	7
3	38/M	Variceal bleeding	PAPS	7	Bleeding	Covered+bare	No	No	No	37.5	14.5	80	17.7	7
4	62/M	Variceal bleeding	HBV	11	Bleeding	Covered+bare	No	No	No	25.4	7.4	65	16.7	8
5	47/M	Variceal bleeding	HBV	18	Bleeding	Covered+bare	Yes^∗∗^	No	Yes	36.7	69	92	15.3	8
6	46/M	Variceal bleeding	HBV	9	Bleeding	Covered+bare	No	No	No	35.6	16.7	61	14.6	6
7	54/F	Variceal bleeding	HBV	8	Bleeding	Covered+bare	No	Yes	No	33.6	26.3	56	15.4	7
8	64/F	Variceal bleeding	HBV	11	Bleeding	Covered	No	No	No	33.8	21.6	64	21.1	8
9	71/M	Variceal bleeding	HCV	12	Bleeding	Covered	No	Yes	Yes	37.2	19.7	120	14.6	8
10	67/M	Variceal bleeding	HBV	11	Bleeding	Covered+bare	No	No	No	36.5	13	63	15.1	6
11	54/M	Ascites	HBV	10	Ascites	Covered+bare	No	Yes	No	36.7	80	79	18.4	9
12	59/F	Variceal bleeding	HBV	9	Bleeding	Covered+bare	No	No	No	31.7	22	50	12.5	6
13	60/M	Variceal bleeding	HBV	13	Bleeding	Covered	No	No	No	40.4	10.7	60	12.2	5
14	41/M	Variceal bleeding	HBV	8	Bleeding	Covered+bare	No	No	Yes	33.6	11	99	19.2	9

^∗^Angioplasty; ^∗∗^bare stent implanted; BCS = Budd-Chiari syndrome; HBV = hepatitis B virus infection; HCV = hepatitis C virus infection; PAPS = primary arterio-portal shunts; PT = prothrombin time.

**Table 2:** Technical data and outcomes.

No.	Age/sex	Technical success	Preprocedure PPG (mmHg)	Postprocedure PPG (mmHg)	Outcomes	Follow-up (months)
1	26/F	Yes	35	15	Asymptomatic	14
2	67/M	Yes	30	14	Death	0
3	38/M	Yes	21	10	Asymptomatic	13
4	62/M	No^∗^	17	9	Asymptomatic	16
5	47/M	Yes	23	14	Asymptomatic	16
6	46/M	Yes	16	8	Asymptomatic	17
7	54/F	Yes	19	10	Asymptomatic	20
8	64/F	Yes	17	10	Asymptomatic	13
9	71/M	Yes	22	14	Asymptomatic	11
10	67/M	Yes	25	15	Asymptomatic	18
11	54/M	Yes	27	16	Bleeding^∗∗^	10
12	59/F	Yes	19	9	Asymptomatic	13
13	60/M	Yes	31	15	Asymptomatic	14
14	41/M	Yes	18	12	Asymptomatic	12

^∗^Created parallel TIPS as alternative, ^∗∗^further TIPS revision confirmed stenosis at proximal end and hence deployed a bare stent.

## Abbreviations

BCS: Budd-Chiari syndrome
ePTFE: Expanded polytetrafluoroethylene
HBV: Hepatitis B virus infection
HCV: Hepatitis C virus infection
HE: Hepatic encephalopathy
IVC: Inferior vena cava
PAPS: Primary arterio-portal shunts
PPG: Portosystemic pressure gradient
PT: Prothrombin time
RA: Right atrium
TIPS: Transjugular intrahepatic portosystemic shunt.
